# Efficient Qualitative and Quantitative Determination of Antigen-induced Immune Responses*

**DOI:** 10.1074/jbc.M116.736660

**Published:** 2016-06-10

**Authors:** Danlin Yang, Lee Frego, Marcio Lasaro, Kristopher Truncali, Rachel Kroe-Barrett, Sanjaya Singh

**Affiliations:** From the Department of Biotherapeutics Discovery, Immune Modulation and Biotherapeutics Discovery, Boehringer Ingelheim Pharmaceuticals, Inc., Ridgefield, Connecticut 06877

**Keywords:** drug discovery, epitope mapping, immunology, kinetics, mass spectrometry (MS), serum, surface plasmon resonance (SPR), vaccine development

## Abstract

To determine the effectiveness of immunization strategies used in therapeutic antibody or vaccine development, it is critical to assess the quality of immunization-induced polyclonal antibody responses. Here, we developed a workflow that uses sensitive methods to quantitatively and qualitatively assess immune responses against foreign antigens with regard to antibody binding affinity and epitope diversity. The application of such detailed assessments throughout an immunization campaign can significantly reduce the resources required to generate highly specific antibodies. Our workflow consists of the following two steps: 1) the use of surface plasmon resonance to quantify antigen-specific antibodies and evaluate their apparent binding affinities, and 2) the recovery of serum IgGs using an automated small scale purification system, followed by the determination of their epitope diversity using hydrogen deuterium exchange coupled with mass spectrometry. We showed that these methods were sensitive enough to detect antigen-specific IgGs in the nanogram/μl range and that they provided information for differentiating the antibody responses of the various immunized animals that could not be obtained by conventional methods. We also showed that this workflow can guide the selection of an animal that produces high affinity antibodies with a desired epitope coverage profile, resulting in the generation of potential therapeutic monoclonal antibody clones with desirable functional profiles. We postulate that this workflow will be an important tool in the development of effective vaccines to combat the highly sophisticated evasion mechanisms of pathogens.

## Introduction

One hundred years after the “magic bullet” proposal by Paul Ehrlich and decades after the establishment of hybridoma technology for producing monoclonal antibodies (mAbs) by Köhler (1) and others (2, 3), antibodies have finally become very attractive therapeutic modalities. The high potency of antibodies and the ability to generate a seemingly unlimited diversity of specificities have led to antibody-based therapies that target many different illnesses and diseases (4–6). The majority of therapeutic antibodies currently available on the market are of mouse origin. Despite the proven success of animal immunizations for over 3 decades, the generation of mAbs for therapeutic purposes is often a labor-intensive and time-consuming process that requires months of work from the initial immunization to the identification of specific hybridoma antibodies. Although other approaches, such as phage display (7) and human memory B-cell immortalization and cloning (8), have been developed for the derivation of fully human mAbs that do not require animal immunization, these methods have shown limited success due to the challenges associated with antibodies of non-immune origins (9). To date, the production of mAbs from immunized rodents remains the most highly used platform for generating high quality therapeutic biologics, because of the natural *in vivo* recombination and affinity maturation of antibodies that occur during the humoral immune response in immunized animals (10–12).

Generating an effective immune response is not only fundamental for therapeutic antibody discovery, but is also crucial for vaccine development in combating infectious disease. Both begin with the host immune response elicited against the foreign antigen/vaccine after immunization. While animal immunization for therapeutic antibody generation requires a mechanism-driven strategy for obtaining antibodies that can demonstrate the therapeutic mode of action prior to hybridoma fusion and/or B-cell recovery efforts, vaccine development emphasizes the stimulation of protective immune responses by the host's immune system that are of sufficient strength and quality to sustain efficient and long term protection (13, 14). The development of an optimal immune response to meet either goal is challenging due to the sophisticated mechanisms that control immune responses (15). The process often involves multiple critical considerations, such as the selection of candidate antigens, choice of adjuvant, antigen/vaccine design, dosage, frequency of application, duration of immunization, injection protocols, etc. (16–18). Immune responses are typically monitored by measuring antibody titers (the relative concentration of antigen-specific antibodies) in the blood samples collected from the immunized hosts over the course of immunization. While many commercial kits and automated systems are available, ELISA remains the most widely used method for antibody titer measurements due to its simplicity and low cost. However, these conventional assays can only partially evaluate immune responses (19, 20), as they cannot accurately reveal data about the binding affinity, specificity, and epitope diversity of polyclonal antibodies, all of which are important for an effective immunization campaign that will lead to the generation of high quality antibody responses with distinct specificities.

To increase the efficiency of our therapeutic antibody generation process to achieve long term success, we developed alternative methods for assessing the quality of polyclonal antibodies in immune sera, surface plasmon resonance (SPR)^2^ and hydrogen deuterium exchange (HDX) coupled with mass spectrometry. We propose that a workflow integrating these sensitive methods into the antibody generation platform can help “engineer” the immune response by providing information on the effectiveness of various immunization methods and by selecting the most appropriate animals for antibody recovery. Access to this information increases the probability of success in recovering antibodies that meet affinity and/or epitope requirements. In this report, we describe the following: the method development and establishment of the detection limits of our techniques, a proof-of-concept study using sera from IL-13 immunized mice, the creation of a workflow named Quality of Antibody Response (QAR), and finally the implementation of this workflow for a therapeutic anti-B-cell activating factor (BAFF) antibody generation campaign to guide the selection of optimal animal donors for hybridoma generation. We demonstrate how these sensitive methods can effectively differentiate the antibody responses among various animals, thereby contributing to the successful recovery of functional antibodies. Beyond its use in therapeutic antibody discovery, this QAR workflow can also be applied to advance vaccine development toward the goal of developing robust vaccines.

## Results

### 

#### 

##### Serum IgG Purification Efficiency

**FIGURE 1.**
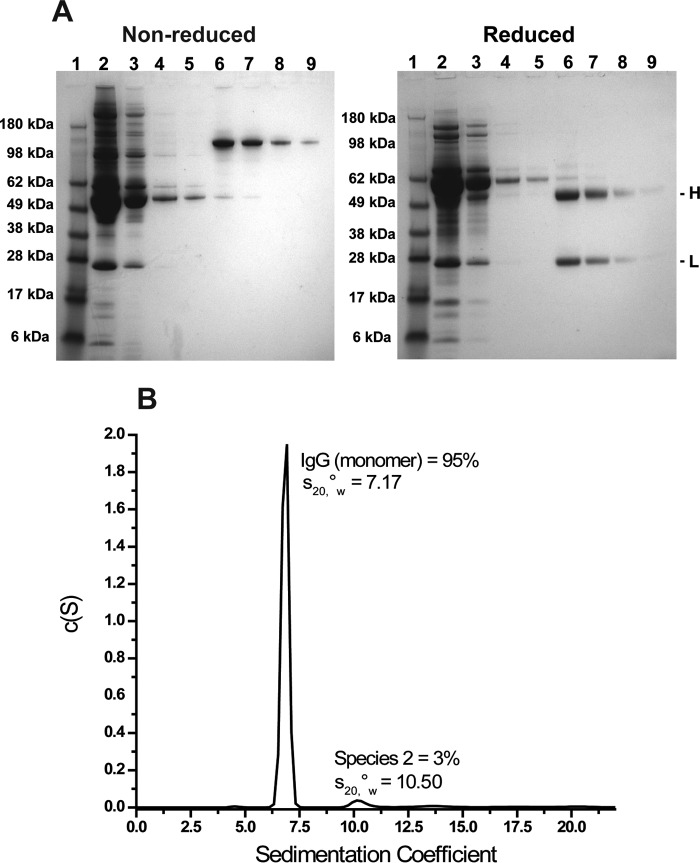
**Evaluation of a small scale mouse serum IgG purification using the PhyNexus automated MEA system.** *A*, SDS-PAGE analysis of samples obtained after various steps of the purification process. Samples maintained under non-reducing (*left*) and reducing (*right*) conditions were run in parallel on individual pre-cast polyacrylamide gels. Markers are in *lane 1*; flow-through in *lane 2*; washes in *lanes 3–5* (DPBS; DPBS + 1 m NaCl; DPBS, respectively), and sequential elutions in *lanes 6–9. H* and *L* denote IgG heavy and light chain bands in the reducing gel. *B*, continuous sedimentation coefficient (*s*) and distribution of purified mouse IgGs in 60 mm sodium acetate, pH 5.0, buffer after AUC. *s*__20,_*_w_*_^0^ denotes the apparent corrected sedimentation coefficient calculated using the buffer's density and viscosity. The 95% peak represents IgG monomers.

To enable analysis by mass spectrometry, serum IgGs were purified using the PhyNexus automated purification system (Table 1). To ensure that the purification process achieved maximal IgG recovery and optimal IgG quality, SDS-PAGE and AUC were used. We used naive serum to investigate the number of elution cycles required for full retrieval of the bound IgGs; SDS-PAGE was used to track the presence of IgGs in the samples from each of 10 consecutive elution steps. We found that IgGs could no longer be detected beyond the 5th elution cycle, suggesting that four elution steps were sufficient (data not shown). Thus, we used four elution steps throughout this study. Non-reducing SDS-PAGE analysis of the material from each of the purification steps showed that the ProPlus (protein A/G) resin effectively captured IgGs from the mouse sera (Fig. 1*A*). Although the remaining flow-through and washing step solutions contained multiple bands, the eluted solutions showed a single band whose size corresponded to the typical IgG molecular mass of ∼150 kDa. This result was confirmed using reducing SDS-PAGE, which revealed the presence of two bands with molecular weights corresponding to those of the heavy and light chains. The IgG bands from the four sequential elution cycles gradually decreased in signal intensity as expected. The pooled eluate, whose total collected volume was ∼2 ml, was concentrated, and its purity and homogeneity were evaluated using AUC. Sedimentation velocity analysis showed that the sample contained 95% monomeric IgGs (Fig. 1*B*).

**TABLE 1:** **Operating conditions used for the affinity purification of mouse serum IgGs in the PhyNexus MEA purification system**

Step	Reagent	Reagent position	Reagent volume	Intake volume	Flow rate	No. of cycles	Delay	Position axis
			μ*l*	μ*l*	*ml/min*		*s*	
Column equilibration	PBS	Plate position 7, row A	1000	900 (920 expelled in the last cycle)	0.5	2	20	2575
Capture	Serum IgGs diluted in PBS	Plate position 3, row A	500	400 (420 expelled in the last cycle)	0.25	10	20	2660
Wash 1	PBS	Position 7, row B	1000	900	0.5	3	20	2575
Wash 2	PBS + 1 m NaCl	Position 7, row C	1000	900	0.5	3	20	2575
Wash 3	PBS	Position 7, row D	1000	900 (920 expelled in the last cycle)	0.5	3	20	2575
Elution 1	30 mm sodium acetate, pH 3.0	Position 7, row E	500	400	0.5	5	20	2575
Elution 2	30 mm sodium acetate, pH 3.0	Position 7, row F	500	400	0.5	5	20	2575
Elution 3	30 mm sodium acetate, pH 3.0	Position 7, row G	500	400	0.5	5	20	2575
Elution 4	30 mm sodium acetate, pH 2.5	Position 7, row H	500	400 (440 expelled in the last cycle)	0.5	5	20	2575
Neutralization (intake)	300 mm sodium acetate, pH 9	Position 8, row B	300	50	6	1	3	2050
Neutralization (expelled)		Step-wise deposition to row E–H		50	2	1	3	

##### Detection Limit of Antigen-specific IgGs in Serum Using SPR

**FIGURE 2.**
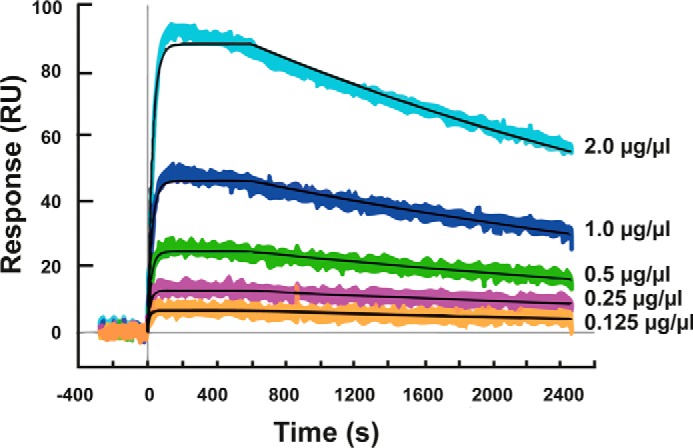
**SPR sensorgrams of human IL-13 binding to mAb DL11 added to mouse serum.** The binding events were recorded in real time upon the injection of human IL-13 at 5 nm over the five antibody surfaces simultaneously. Each sensorgram represents a different concentration of DL11: 2.0 μg/μl (*light blue*), 1.0 μg/μl (*dark blue*), 0.5 μg/μl (*green*), 0.25 μg/μl (*purple*), and 0.125 μg/μl (*orange*). The *black lines* that overlay the response curves represent the local fit obtained by applying the Langmuir 1:1 kinetic binding model. The binding affinity was determined to be 24 (±8) pm.

The concentration-dependent binding of IgG to human IL-13 was demonstrated by SPR using samples containing naive serum with various amounts of DL11 added. Because of the high concentration of endogenous IgGs, the serum samples were diluted 1000-fold prior to protein A/G capture. The total IgGs, which included both endogenous IgGs and DL11, were captured at a density of ∼2000 RU. As expected, the binding response decreased with decreasing DL11 concentrations in the serum (Fig. 2). The affinity of DL11 was determined to be ∼24 (±8) pm, and saturation was achieved at 5 nm human IL-13. There was no difference in the maximal response level when 10 and 100 nm human IL-13 solutions were injected over identical IgG-containing surfaces (data not shown). The concentration limit at which antigen-specific IgGs were detected in serum was determined to be 250 ng/μl, as signals <10 RU are considered to be background noise in this system. Using Equations 1 and 2, described under “Experimental Procedures,” the percentage of DL11 antigen-specific IgGs among the total IgGs was calculated for each reconstituted sample. As shown in Table 2, the lower limit of 250 ng/μl corresponded to a concentration that was equivalent to ∼10% of the total IgGs being human IL-13-specific DL11.

**TABLE 2:** **Quantification of mAb DL11 in mouse serum** NA means not applicable.

Concentrations of DL11 added to serum	Total IgG captured	Theoretical *R*_max_	Experimental *R*_max_	Human IL-13-specific IgGs
	*RU*	*RU*	*RU*	
2.0 μg/μl	2547	209	93	45%
1.0 μg/μl	2164	177	49	28%
0.5 μg/μl	2168	178	27	15%
0.25 μg/μl	2016	165	14	8%
0.125 μg/μl	1907	156	9	6%
0.063 μg/μl	1909	157	No binding	NA
0.031 μg/μl	2044	168	No binding	NA
0.016 μg/μl	1990	163	No binding	NA

##### Detection Limit of Antigen-specific IgG in Serum Using HDX-LC/MS

**FIGURE 3.**
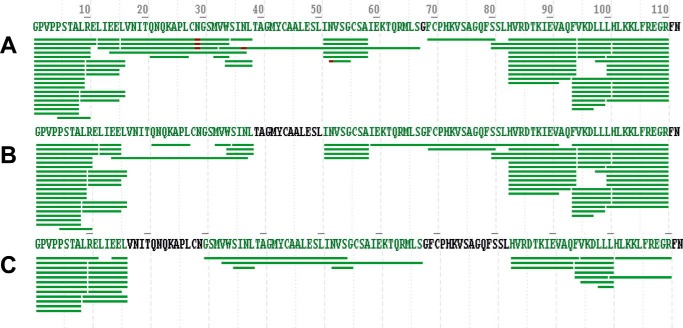
**Human IL-13 sequence coverage from on-line pepsin digestion.** Human IL-13 prepared at 4 μm (*A*), 2 μm (*B*), and 1 μm (*C*) in PBS was digested with pepsin and the resulting peptides, which exhibited different recorded retention times, were analyzed by PMi Byonic, resulting in sequence coverage that was IL-13 concentration-dependent: 98% (4 μm), 88% (2 μm), and 73% (1 μm). Each *bar below* the sequence represents an individual peptide. The residues in *green* indicate sequences covered by the identified peptides, and those in *black* represent uncovered regions. The residue in *red* (*N*) in the identified peptides was found to be highly deamidated.

To determine the IgG-binding epitope on the antigen, the purified IgGs were subjected to HDX coupled with LC/MS. This method compares deuterium exchange levels at different regions of the antigen in the presence and absence of antibody and identifies those that are protected by the IgGs. First, a mock experiment using different concentrations of human IL-13 in the absence of IgGs was performed to determine the optimal concentration of protein that was required to ensure full sequence coverage of the protein. The results revealed that a concentration of 4 μm yielded the highest sequence coverage (98%) (Fig. 3). Thus, this concentration was used in the subsequent experiments using IgG samples.

**FIGURE 4.**
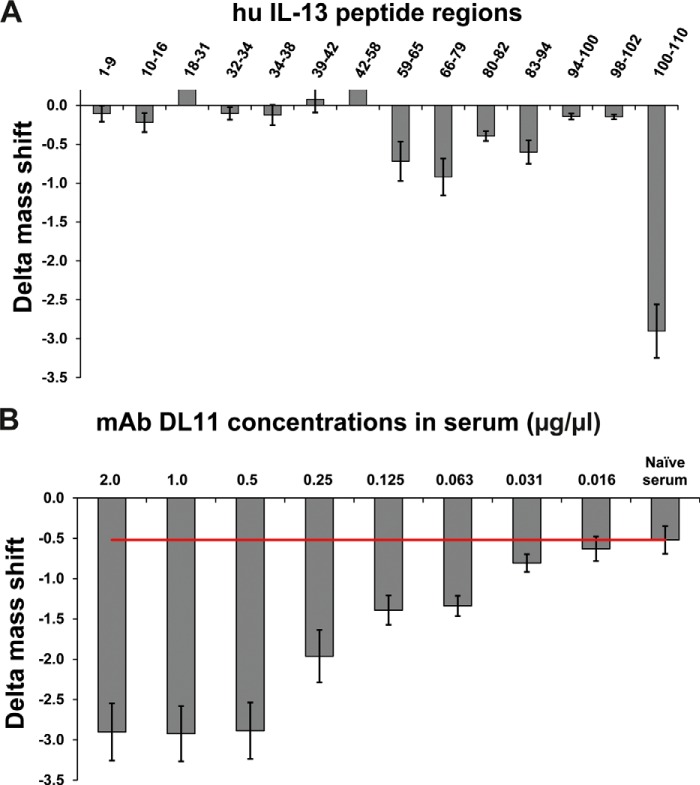
**Deuterium uptake in human IL-13 in the presence of mAb DL11.** The mass shifts were calculated based on the differences in deuterium uptake in human IL-13 in the presence and absence of DL11 over reaction periods that spanned 60, 120, and 240 s. The more negative Δ mass shifts were correlated with stronger protection by DL11 binding. *A*, epitope mapping of DL11 based on mass shifts in human IL-13 peptides. *B,* DL11 concentration-dependent mass shift resulting from the protection of human IL-13 peptide region 100–110 (LHLKKLFREGR). The *red line* represents the background signal from the naive serum. Results represent mean and S.E. (*error bars*) obtained from three time points.

The epitope mapping experiment was performed by incubating the purified IgGs with human IL-13 as described under “Experimental Procedures.” Deuterium uptake was compared in samples containing human IL-13 in the absence and presence of DL11. Because hydrogen has a mass of 1.008 Da and deuterium has a mass of 2.014 Da, the replacement of hydrogen in a protein with deuterium induces a shift in mass of ∼1 Da. Because antibody binding inhibits deuterium exchange in the epitope regions, peptides exhibiting shifts in mass are associated with antibody epitopes. As shown in Fig. 4*A*, a large shift in mass in comparison with the control was observed in the peptide encompassing residues 100–110 in human IL-13, indicating that this region was protected by DL11. Therefore, the shift in mass of this peptide was monitored in the limit of detection study, in which a fixed amount of human IL-13 was incubated with samples containing titrated concentrations of purified DL11. The endogenous IgGs purified from naive serum were used as a negative control to establish the background signal.

The results shown in Fig. 4*B* indicated that the observed shift in mass corresponding to the reduced deuterium incorporation in human IL-13 was IgG concentration-dependent, as expected. The limit of detection was determined to be 31 ng/μl, indicating that this was the minimum IgG concentration required to yield a detectable amount of deuterium protection.

##### Proof-of-Concept Study Using Sera from Immunized Mice

**FIGURE 5.**
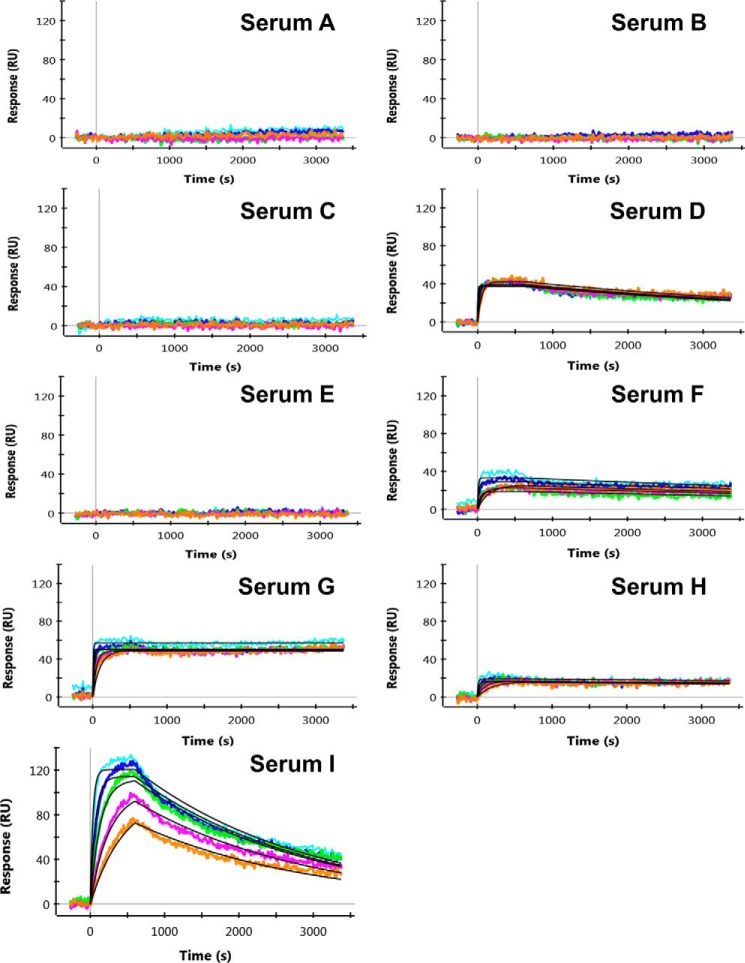
**Characterization of binding interactions between mouse serum IgGs and human IL-13.** The binding interactions were monitored over 10-min association periods followed by 45-min dissociation periods. The binding response curves were obtained by measuring the binding of human IL-13 to IgGs captured on the biosensor surface that were titrated by 2-fold dilutions as follows: 100 nm (*light blue*); 50 nm (*dark blue*); 25 nm (*green*); 12.5 nm (*purple*); and 6.25 nm (*orange*). Kinetic fit was performed on serum samples D and F–I using the 1:1 Langmuir kinetic binding model in the ProteOn analysis software, as illustrated by the *black lines* that overlay the response curves.

A total of nine ∼100-μl serum samples from mice of various strains that were immunized with human IL-13 were evaluated using our SPR and HDX-LC/MS methods. Each of the serum samples had previously been characterized as a “binder” by a single point ELISA analysis. As shown in the results summary (Table 3), our analysis showed that the serum samples contained different percentages of human IL-13-specific antibodies, and exhibited significantly different average apparent affinities (Fig. 5). Among the nine serum samples, four (samples A–C and E) showed no human IL-13 binding at the highest tested concentration (100 nm). Of the other five samples that showed binding, the apparent affinities ranged from single/double digit picomolars to single digit nanomolars, as shown by the SPR binding data. The difference in affinities was influenced by variations in both *k_a_* and *k_d_* values. Although serum samples D and F–H exhibited faster on-rates and slower off-rates, and hence lower *K_D_* values or higher affinity interactions than those of serum I, serum I exhibited 3–6-fold higher amounts of human IL-13-specific IgGs than the others, based on its larger experimental *R*_max_ values.

**TABLE 3:** **Evaluation of kinetic binding and epitope mapping of serum antibodies from animals immunized with human IL-13**

Sample ID	*k_a_* (1/ms)	*k_d_* (1/s)	Apparent *K_D_*	% human IL-13 specific IgG	Binding epitope
			*pm*		
Serum A			No binding observed		None
Serum B			No binding observed		None
Serum C			No binding observed		None
Serum D	3.47E+6	1.88E−4	54	∼23%	34–38, 66–79, 101–110
Serum E			No binding observed		None
Serum F	1.42E+6	1.21E−4	85	∼12%	101–110
Serum G	2.57E+6	<1.0E−6	<20	∼26%	9–16, 34–38, 51–58, 66–79, 83–94, 101–110
Serum H	1.04E+6	3.92E−5	38	∼10%	1–9, 9–16, 83–94, 101–110
Serum I	3.00E+5	4.43E−4	1480	∼66%	9–16, 83–94, 101–110

**FIGURE 6.**
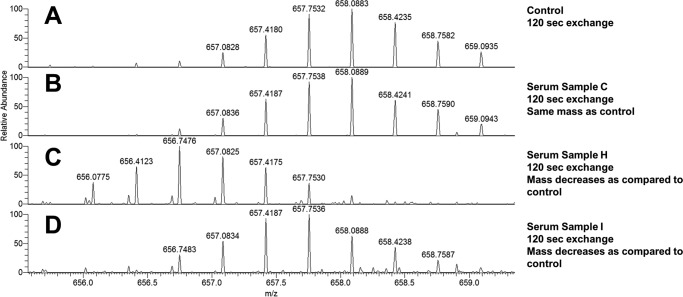
**Comparison of mass shifts in human IL-13 following HDX LC/MS analysis using purified IgGs from three different anti-IL-13 sera.** Purified IgGs were incubated with human IL-13, followed by exposure to deuterium for 120 s, digestion with pepsin, and LC/MS analysis. *A–D,* MS analysis of the region encompassing residues 95–110 in human IL-13. *A*, human IL-13 incubated in the absence of IgG. *B*, human IL-13 incubated with purified IgGs from serum C. *C*, human IL-13 incubated with purified IgGs from serum H. *D,* human IL-13 incubated with purified IgGs from serum I. *Peaks* represent the isotopic ion distributions. A shift of the isotope distribution to the left in comparison with the control sample indicates IgG protection of the region from deuterium exchange.

**FIGURE 7.**
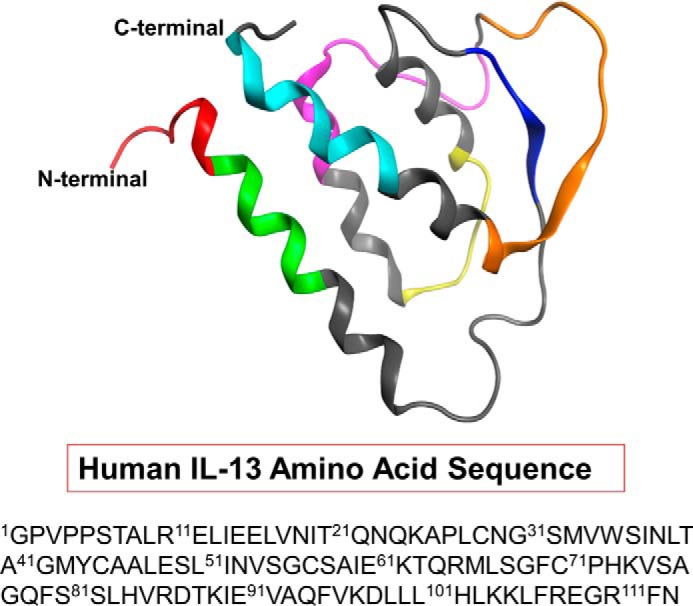
**Epitope diversity observed in the IgGs from the serum of mice immunized with human IL-13.** The sequence of human IL-13 was obtained from NCBI (www.ncbi.nlm.nih.gov, accession number AAK53823.1). The crystal structure was obtained from Protein Data Bank (Protein Data Bank code 1IJZ (39)). The different colors represent each of the identified epitopes and encompassed the following residues: 1–9 (*red*); 9–16 (*green*); 34–38 (*blue*); 51–58 (*yellow*); 66–79 (*magenta*); 83–94 (*orange*); and 101–110 (*cyan*). *Gray* indicates the absence of an antibody response toward the region.

In addition to observing differences in the antigen-specific apparent affinities and quantities of polyclonal IgGs among the nine serum samples, we found that the IL-13 binding epitopes among the serum samples were also different. As shown in Fig. 6, the IgGs from serum H (*panel C*) protected the region encompassing residues 95–110 from deuterium exchange, indicating that this serum sample contained IgGs that were specific to this region. Similarly, the IgGs from serum I (Fig. 6, *panel D*) also showed protection on the same region as that of serum H, with a relatively less extensive mass shift that was likely due to weaker affinity to human IL-13. In contrast, the IgGs from serum C (Fig. 6, *panel B*) showed no protection at this region, as the exchange observed there was equivalent to that of the control, containing no IgGs (*panel A*), suggesting that either this serum sample contained no IgGs targeting this region or that the level of IgGs present was below the detection limit of the method (31 ng/μl). The results obtained using both SPR and HDX LC/MS were consistent, in that the non-binders showed no binding of any region in human IL-13. For those samples that exhibited epitope protection, a diverse range of epitopes were identified. As depicted in Fig. 7, antibody responses directed toward various helix and loop regions of the human IL-13 protein were detected. Of the 112 amino acids in human IL-13, 63 were shown to be protected, indicating that 56% of the sequence elicited antibody responses. While serum G exhibited the most diverse responses, serum F, which contained antibodies that were exclusively directed against the C terminus of the protein (Table 3), exhibited the least diverse response.

##### Integration of the Workflow Process into Our Antibody Generation Platform

**FIGURE 8.**
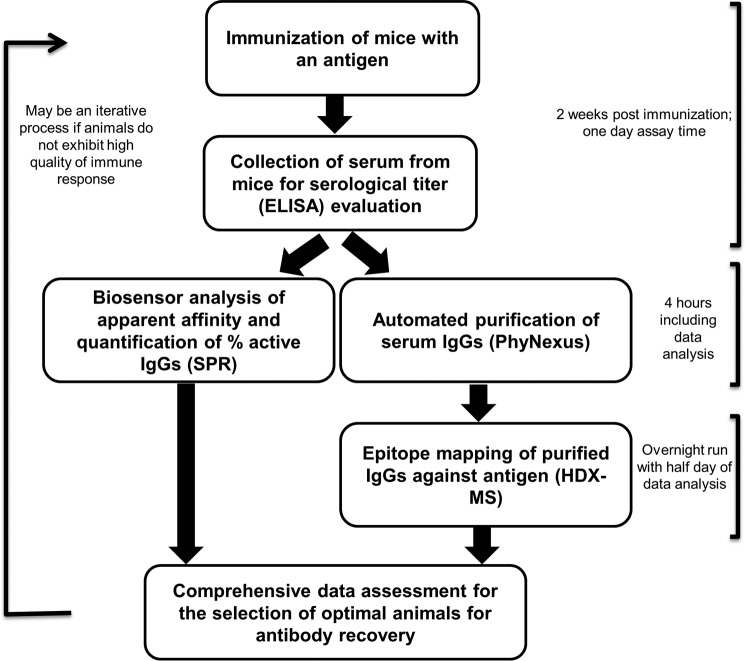
**Flowchart illustrating the QAR workflow and the time frames for each of the methods.**

Given the success of our methods in differentiating the antibody responses among different immunized animals, we integrated the QAR workflow into our antibody generation platform to help guide decision-making in future immunization campaigns. The flowchart shown in Fig. 8 indicates the time frames of the QAR workflow components. Approximately 2 weeks after the immunization of animals with antigen or antigen/adjuvant mixtures, after the induction of an effective antibody response, serum samples are evaluated by ELISA to determine the presence and titer of antibodies. If the titer results are positive, the serum samples are directly analyzed by SPR to determine their apparent binding affinities and to quantify the percentage of antigen-specific IgGs. At the same time, the remaining volume of the serum samples (∼99 μl) is subjected to IgG purification using the PhyNexus automated purification system. While the SPR method can measure six serum samples at a time (six parallel sample injections based on the 6 × 6 multiplex configuration) with a protocol that typically includes multiple binding cycles, the PhyNexus MEA system can purify 12 samples at a time with an estimated run time of 2.5 h. Multiple MEA systems can be used in parallel to analyze more than 12 samples simultaneously. Taking into account the post-purification sample preparation time (enrichment and buffer-exchange steps), purified IgG samples can be ready for epitope mapping within 4 h. Meanwhile, analysis of the SPR data can provide early information on the affinity and quantity of the antibodies. After sample and reagent preparation, the HDX LC/MS experiment is performed overnight using an automated protocol that accurately controls both the deuterium exchange and quenching reaction times for each sample. Deuterium exchange, involving the sequential analysis of three time points, is performed and completed by the next day. Because of the large amount of data collected, the MS data analysis requires about a half-day of data processing using two in-house-derived proprietary software applications. Thus, a comprehensive data package can be generated within 2 days that provides useful feedback for guiding the immunization approach. The workflow can be applied at any point during the immunization campaign to track the development of antibody responses.

##### Implementation of the QAR Workflow to Guide Antibody Recovery

The QAR workflow was integrated into the BAFF antibody generation campaign, and its performance was evaluated. Similar to the anti-IL-13 sera analysis, the anti-BAFF sera analysis (Table 4) revealed differences in antibody quality among the 12 serum samples. In addition to analyzing antibody binding to the human antigen that was used for immunization, we also analyzed antibody binding to the cynomolgus (cyno) monkey protein using SPR. Even though the human and cyno-BAFF proteins share 97% sequence identity, serum samples from mice P5804-01 and P5804-02 exhibited *K_D_* values for cyno-BAFF that were five times those for human BAFF, indicative of weaker interactions. We analyzed the binding epitopes of the various antibodies, focusing on the antibody responses to the regions in human BAFF that are known to be important for receptor interactions. Our in-house analysis indicated that regions encompassing residues 17–31, 68–90, 128–137, and 137–145 are important for function (data not shown). Therefore, we focused our attention on IgGs that were found to target these regions. Among the 12 serum samples, only five (P5801-01, P5801-02, P5811-01, P5821-01, and P5821-02) showed binding responses toward at least one of the prioritized epitope regions. Although four of these samples exhibited similar double digit picomolar or better affinities and less than ∼2-fold shifts in binding to cyno-BAFF, the serum sample from mouse P5801-02 contained the highest amounts (>30%) of human BAFF and cyno-BAFF-specific IgGs. This mouse was subsequently selected for hybridoma fusion, and analysis of the subcloned monoclonal antibodies (Table 5) revealed that they exhibited low picomolar EC_50_ binding to human BAFF. In addition, blockade of receptor binding was confirmed, indicating that the selection of mice based on the epitope mapping of serum IgGs is an effective strategy.

**TABLE 4:** **Evaluation of cross-species binding affinity and epitope mapping of serum antibodies from mice of A/J strain immunized with human BAFF** NM means not measured.

Mouse ID	Vaccine	Apparent *K_D_* to hu BAFF	% human BAFF-specific IgG	Apparent *K_D_* to cyno-BAFF	% cyno BAFF-specific IgG	Epitope (17–38)	Epitope (68–90)	Epitope (128–136)	Epitope (137–145)
		*pm*		*pm*					
P5801-01	Particle vaccine A	38	23%	117	25%	+	−	−	−
P5801-02	Particle vaccine A	23	31%	53	36%	+	−	−	−
P5804-01	Particle vaccine A	53	12%	263	11%	−	−	−	−
P5804-02	Particle vaccine A	66	8%	345	6%	−	−	−	−
P5811-01	Particle vaccine B	13	18%	26	19%	+	−	−	−
P5811-02	Particle vaccine B	39	24%	90	27%	−	−	−	−
P5814-01	Particle vaccine B	Insignificant binding	3%	Insignificant binding	3%	NM	NM	NM	NM
P5814-02	Particle vaccine B	<20	10%	68	11%	−	−	−	−
P5821-01	Particle vaccine C	<20	20%	<20	23%	+	+	+	-
P5821-02	Particle vaccine C	<20	18%	<20	20%	+	−	−	−
P5824-01	Particle vaccine C	<20	6%	<20	6%	−	−	−	−
P5824-02	Particle vaccine C	<20	8%	<20	9%	−	−	−	−

**TABLE 5:** **Characterization of anti-BAFF subclones recovered from mouse P5801-02**

Clone name	Subclone ID	Concenration	Subclass	mAb-BAFF binding EC_50_	TACI blocking IC_50_
		μ*g/ml*		*pm*	*pm*
715D12	A2	30	IgG1	<10	300
715D12	A7	30	IgG1	<10	300
727B10	A8	10	IgG1	<10	500
727B10	B6	15	IgG1	<10	500
730B7	A3	15	IgG1	<10	500
730B7	C5	20	IgG1	<10	500
741A6	B11	5	IgG1	20	>2000
741A6	E1	10	IgG1	15	>2000
743F3	A12	10	IgG1	<10	700
743F3	B8	15	IgG1	<10	700
745F10	A2	<1	ND*^a^*	ND	ND
745F10	D10	<1	ND	ND	ND
748A8	B11	5	IgG1	<10	400
748A8	B12	5	IgG1	<10	400
748B9	C1	10	IgG1	<10	400
748B9	E8	10	IgG1	<10	400
751D4	B9	10	IgG1	<10	500
751D4	H4	30	IgG1	<10	600
759F7	G10	10	IgG1	<10	600
759F7	H8	30	IgG1	<10	400
764C1	B2	5	IgG1	25	3000
764C1	D8	25	IgG1	10	1000
765B9	B9	15	IgG1	<10	300
765B9	H4	20	IgG1	<10	300

*^a^* ND means not determined.

## Discussion

To increase the probability of success in recovering criteria-meeting functional antibodies from immunization campaigns, we explored the feasibility of applying methods that are typically used later in the antibody identification process, namely SPR and mass spectrometry, to characterize the antibodies in sera from immunized mice. The profiling of immune responses using the standard ELISA method to measure the effectiveness of immunization and vaccination has been widely applied and reported (21–24). Here we used SPR instead, because it has several advantages over the traditional ELISA method. SPR is a label-free technique that can be performed in real time, but most importantly, it can provide highly precise measurements for determining association and dissociation rate constants (25–27), which cannot be achieved by ELISA. The application of SPR for analyzing complex biological samples has been demonstrated in several reports (28–30), although its use for these applications is still rare compared with other immunoassays. Another advantage of using SPR for both quantitative and qualitative evaluation of IgGs in serum is the ease of including a “purification” step in which IgGs are captured by protein A/G onto the sensor surface. Protein A/G has affinity for mouse IgG isotypes 1, 2a, 2b, and 3. Therefore, the use of this reagent ensures that the antigen affinities of all the antibodies in the mouse serum will be effectively evaluated and that the resulting binding signal is highly specific for the IgGs. Furthermore, the amount of antigen-specific IgGs present in the serum can be quantified through calculations from the *R*_max_ values. According to our results using reconstituted samples, the detection limit for high affinity, low picomolar binding antibodies in serum using SPR was ∼250 ng/μl.

Epitope mapping to elucidate both the specificity of the antibody responses and their diversities is crucial for obtaining antibodies with a desirable mechanism of action (31, 32). Traditionally, the antigen-antibody contact surfaces have been determined by methods such as high resolution x-ray crystallography and site-directed mutagenesis (33, 34). However, these methods are not applicable for our purpose, because our samples contain antibody mixtures. In addition, the amount of material available for analysis and the time frame for acquiring epitope information are both limited. In search of a suitable technique, we initially explored peptide microarray (35, 36), in which a library of linear synthetic peptides is used to screen for IgG-binding epitopes. Samples containing mAbs with known binding epitopes, as well as sera from both naive animals and animals immunized with human IL-13, were tested using these methods. However, we were unable to identify specific epitopes for either the mAb or the antibodies present in the anti-IL-13 sera (data not shown). Regardless, these findings provided two important insights. First, we learned that the binding epitopes of the control mAbs were conformational, rather than linear, and second, we learned that the presence of serum proteins probably interfered with the methods. Therefore, we considered alternative techniques that could facilitate the identification of native protein conformational epitopes in solution with high resolution.

Ultimately, we selected HDX coupled with LC/MS due to its low material consumption, relatively high throughput via robotic automation, and high sensitivity. The combination of hydrogen deuterium exchange with mass spectrometry has proven to be increasingly powerful for characterizing protein conformations, structural dynamics, and protein-protein interactions (37). Even though HDX is an established method and had successfully been applied in-house to map epitopes of purified mAbs at high concentrations, several challenges exist for the evaluation of IgGs in serum. Therefore, additional method development was required to improve the feasibility of this approach. One major challenge is the matrix effects from serum, which could produce significant interference if injected directly into the instrument. To address this issue, we developed a small scale affinity purification protocol using the PhyNexus system to purify IgGs from the serum using protein A/G columns. This platform was selected because of the availability of system automation and its relatively high throughput, enabling the purification of 12 serum samples simultaneously. The purification scheme was optimized to achieve maximal IgG recovery and purity, and our results showed that highly pure IgGs could be obtained from serum samples. The second challenge involved the sensitivity of the method for detecting antibody epitopes from a mixture of IgGs, each of which is present at a different concentration. To improve the sensitivity, several modifications were made in the sample preparation, including the use of concentrators to increase the IgG concentration and pH adjustment via buffer exchange to further optimize the deuterium exchange efficiency. With the incorporation of these additional sample-processing steps, it was important to determine that the IgGs were recovered at sufficient concentrations for signal detection. An experiment in which a high affinity mAb with known binding epitopes was evaluated at different concentrations showed that the sensitivity of the method was ∼31 ng of antigen-specific IgG per μl of serum. This high sensitivity can facilitate the detection of less abundant IgGs exhibiting unique epitopes, maximizing the diversity of antibodies that can be recovered.

After establishing the highly sensitive SPR and HDX LC/MS methods for detecting antigen-specific antibodies in serum, we tested their performance using sera from immunized mice. Nine serum samples collected from various strains of mice, which were immunized with human IL-13 using different protocols, were analyzed with these methods in a “proof-of-concept” study. These samples were previously classified as binders by single point ELISA analysis. As anticipated, the results of these analyses allowed us to differentiate the antibody responses among the immunized mice with respect to apparent antigen affinity, amount of antigen-specific activity, and epitope diversity. To inhibit IL-13 function, the development of high affinity anti-IL-13 antibodies that target different epitopes of the IL-13 protein, resulting in either the disruption of IL-13 and IL-13 receptor binding or the disruption of IL-13 and IL-4 α-receptor binding, is required (38). Our analysis of the nine different anti-IL-13 mouse sera revealed important binding affinity and epitope information that allowed us to differentiate the quality of the antibody responses. Although five of the serum samples were found to contain antibodies that bound to the surface of IL-13 that interacts with the IL-13 receptor (epitope 101–110), two sera (D and G) contained additional antibodies that bound to the surface of IL-13 that interacts with the IL-4 α-receptor (epitopes 67–69 (D) and epitopes 51–58 and 66–79 (G)). To maximize the recovery of functional antibodies, serum G therefore would be the best choice for subsequent antibody generation, as it contained high affinity antibodies that could target both mechanisms of action. Although hybridoma fusions were not performed to assess the recovery of functional monoclonal antibodies targeting human IL-13, they were performed to track and assess the antibodies generated for the BAFF program (see below).

During development of the QAR workflow, a top priority was to ensure that the time frame for generating the data package was short enough to enable timely decision-making. As reflected by our selected methods, throughput was an essential consideration. According to our assessment, the complete data package can be compiled within 2 business days, due in large part to the automation of our platform methods (multiplex SPR array, tip column-based walk away serum purification, and liquid-handling HDX reaction control), minimizing the need for extensive manual work. Furthermore, with the implementation of in-house MS software programs for automating the HDX data analysis (beyond the scope of this paper), the entire workflow process can be efficiently executed and can support additional iterations if necessary. As shown in Tables 4 and 5, we used the QAR workflow to guide the development of therapeutic anti-BAFF antibodies, and we demonstrated that the data package contributed to the successful retrieval of functional anti-BAFF antibodies in a timely manner. We focused on the identification of immunized animals that produced high affinity antibodies that targeted well defined epitopes for use in hybridoma fusions. The comprehensive data package generated again confirmed that our methods can provide clear differentiation among the antibody responses in various animals. The finding that our methods consistently provided in-depth and timely information for directing the development of high quality antibodies suggests that they will have widespread utility in guiding therapeutic antibody production in the future.

In summary, we have demonstrated that a combination of sensitive methods can be integrated into the antibody generation platform to help identify animal hosts exhibiting high affinity antibody responses and maximal epitope diversities. The combination of SPR and HDX LC/MS enables the comprehensive quantitative and qualitative evaluation of antibodies present in serum samples. While SPR analysis provides antibody affinity information and antigen-specific antibody quantification, MS analysis provides information about the structural interactions between serum antibodies and their antigens. Thus, these are complementary methods that reveal unique information that cannot be obtained using conventional methods. The proposed workflow process can also be executed at a speed that the biopharmaceutical industry demands to discover novel epitopes for new therapeutic modes of action. In addition, the QAR workflow can be useful in guiding the development of robust vaccines. The described methods can be used to monitor the development of novel immunogens, vaccine preparations, or delivery systems, at the early stages of development, to help predict their efficacy and safety. To our knowledge, this is the first report that demonstrates how the described methods can be implemented to produce in-depth information that distinguishes antibody responses and guides the production of therapeutic antibodies. We intend to follow up with additional studies to demonstrate the utility of this workflow in future therapeutic antibody and vaccine discovery programs.

## Experimental Procedures

### Antigens and Antibodies

Recombinant human IL-13, derived from *Escherichia coli*, was purchased from R&D Systems (Minneapolis, MN). Mouse anti-IL-13 mAb clone DL11, derived from a previous campaign, was produced by transient expression in CHO cells and purified using Mabselect SuRe-based affinity chromatography (GE Healthcare). The purified mAb was formulated in 60 mm sodium acetate, pH 5.0, buffer. To determine the detection limits of our methods, eight 100-μl naive mouse serum samples containing different antibody concentrations ranging from 2 μg/μl to 15.6 ng/μl were prepared by serial dilution. Recombinant human BAFF with a His tag and its receptor TACI-human-Fc chimeric protein were purchased from R&D Systems. Anti-BAFF mAb supernatants were produced using hybridomas generated using spleen cells from mouse P5801-02, followed by cloning and subcloning using limiting dilution. Antibody isotypes were determined using the mouse IsoStrip^TM^ monoclonal antibody isotyping kit (Santa Cruz Biotechnology, Dallas, TX).

### Mouse Sera and Immunizations

Naive mouse pooled serum (0.1 μm filtered) from mixed strains was purchased from BioreclamationIVT (Westbury, NY). Serum samples from nine mice of different strains (BALB/c, C57BL/6, and Swiss Webster) immunized with human IL-13 were stored at −20 °C and used for the proof-of-concept analysis. Mice of various MHC haplotypes were immunized twice with human BAFF. Serum samples were obtained a week after the second immunization and stored at −20 °C. After 10–12 days, the mice were boosted with BAFF, and the spleens were harvested for hybridoma fusion using standard techniques.

### Characterization of Anti-BAFF Hybridoma Supernatants

Hybridoma screening was carried out with Luminex bead-based assays using human BAFF. Positive candidates were cloned by limiting dilution. To measure the EC_50_ value of the mAb-BAFF interactions, magnetic Luminex beads coated with human BAFF were incubated with a dilution series of the mAbs for 1 h at 20 °C. After the mAb-BAFF-bound beads were washed, they were incubated with a biotinylated anti-mouse Fc-specific antibody (1 μg/ml) for 1 h and then incubated with streptavidin-phycoerythrin (4 μg/ml) for another 30 min for signal detection. The anti-BAFF hybridoma subclones were further characterized by their ability to block BAFF-TACI interactions (IC_50_). In this assay, the BAFF-coated beads were first incubated with samples of diluted mAbs for 1 h at 20 °C. TACI-human Fc (5 ng/ml) was then added, and the mixtures were incubated for 2 h, followed by the addition of biotinylated anti-human Fc-specific antibody (1 μg/ml) for 1 h. TACI binding was then detected after a 30-min incubation with streptavidin-phycoerythrin (4 μg/ml).

### Purification of Mouse IgGs from Serum

#### 

##### PhyNexus Automation System and Setup

Mouse antibodies were purified in a fully automated 12-channel PhyNexus MEA purification system (PhyNexus, Inc.) using PhyTip columns packed with 160 μl of ProPlus resin (Part no. PTR41-1607) in 1-ml disposable pipette tips. The ProPlus resin is a protein A-derived ligand that has attributes of both protein A and G, with binding specificities for both mouse IgG_1_ and IgG_2a_ isotypes. Mouse serum samples were diluted in 500 μl of PBS and then transferred into the individual wells of a 96-deep well plate. In addition to the sample plate, two 96-well reagent plates containing the washing, elution, and neutralization buffer for each of the purification steps were also prepared. Although the sample plate was placed in position 3 on the MEA instrument platform and kept at room temperature, the reagent plates were placed in positions 7 and 8 on top of a chiller to maintain the temperature at ∼4–6 °C. A number of PhyTip columns corresponding to the number of samples were transferred into row 1 of an empty 96-well pipette tip box located at position 1. Similarly, the same number of standard 1-ml Rainin LTS pipette tips were transferred into row 2 of the same box for use in the last neutralization step, to transfer the basic solution to the eluent.

After the pre-packed PhyTip columns and reagent plates were loaded onto the MEA instrument platform, the mouse antibodies were isolated using a method programmed with PhyNexus MEA software (version 1.0.17) consisting of the following steps: 1) pre-wash and equilibration of the resin with PBS; 2) capture of IgGs from the diluted 500-μl mouse serum samples with the ProPlus affinity resins; 3) washes with PBS and PBS plus 1 m NaCl to remove other serum proteins; 4) elution of bound IgGs from the resins with 30 mm sodium acetate, pH 3.0, three times, followed by one elution with 30 mm sodium acetate, pH 2.5, to ensure the full removal of any remaining bound IgGs from the resins; and 5) neutralization using 10% (by volume) of 300 mm sodium acetate, pH 9.0, to bring the final formulation to 60 mm sodium acetate, pH 5.0. The details of the purification steps with the respective operating configurations included in the protocols are listed in Table 1. In Table 1, one cycle represents one intake and one expulsion of solution through the ProPlus resin at a speed defined by the flow rate. The flow rate for capturing was maintained at 0.25 ml/min to ensure sufficient contact time between the serum sample and the resins. “Delay” is the delay time between each intake and expulsion step to allow for contact with the solution. The position axis is the distance from the tip to the bottom of the plate; these values were pre-set using the settings recommended by the manufacturer.

##### Detection of IgGs by SDS-PAGE

For protein detection, the samples were analyzed under reducing and non-reducing conditions using NuPAGE 4–12% BisTris gels in NuPAGE MOPS SDS running buffer with the Novex XCell SureLock mini-cell system (Invitrogen) at 200 V for 35 min. The gels were stained using Instant Blue Coomassie-based solution (Expedeon, San Diego). Images of the gels were obtained using a CanoScan 9000F scanner.

##### Quality Evaluation of the Purified IgGs by Analytical Ultracentrifugation (AUC)

The quality of the purified IgGs was evaluated by sedimentation velocity experiments using absorbance optics on a Beckman XLI analytical ultracentrifuge (Beckman Coulter, Inc.). The concentrated IgGs from the elution pools were diluted in 60 mm sodium acetate, pH 5.0, to 0.4 mg/ml, and then 400 μl of each was loaded into the sample chamber, whereas buffer was loaded into the reference chamber of an AUC cell that was assembled with standard double-sector centerpieces and quartz windows. The experiment was conducted at 20 °C using an An60Ti four-hole rotor spinning at 40,000 rpm. The sedimentation process was monitored by collecting absorbance data at 280 nm using the XL-I operating software. The collected data were analyzed using the continuous *c*(*s*) distribution model in the SEDFIT software (version 12.1c) to provide the distribution of sedimentation coefficients. The *s* values obtained with the *c*(*s*) distribution in sodium acetate buffer were converted to *s*__20,_*_w_*_^0^ value with SEDNTERP (version 1.09) using the measured density and viscosity of the buffer.

### Apparent Affinity Analysis and Quantification of Antigen-specific IgGs by SPR

#### 

##### Immobilization of Protein A/G

The biosensor experiments were performed at 25 °C using a ProteOn XPR36 instrument (Bio-Rad). After pre-conditioning, a GLM chip docked into the machine with SDS, NaOH, and NaCl across six channels in both directions, and protein A/G was amine-coupled onto the chip surface using a standard coupling protocol with the following steps. 1) The six individual flow channels were activated in parallel by injecting a freshly mixed solution of 0.2 m *N*(3-dimethylaminopropyl)-*N′-*ethylcarbodiimide in 0.05 m *N*-hydroxysuccinimide. 2) Protein A/G prepared at 50 μg/ml in sodium acetate, pH 4.5, was immobilized on the activated channels. 3) The excess reactive esters were deactivated with 1 m ethanolamine. Each step was performed at a flow rate of 30 μl/min for 5 min. Protein A/G at a high surface density (5000 response units (RU)) was coupled onto the chip surface to capture the serum IgGs and characterize the binding affinities.

##### Characterization of Serum IgG-Antigen Binding Kinetics

A small volume of each serum sample (1 μl) was diluted 1000-fold into the PBS/Tween/EDTA running buffer. Six diluted samples were then injected simultaneously over the six available vertical channels at a flow rate of 25 μl/min, during which the IgGs were captured by the protein A/G. The capture time was monitored to achieve a high antibody surface density (2000–3000 RU). Because of the high abundance of mouse IgGs in the serum samples, a capture time of ∼120–150 s was sufficient to reach the targeted density level. Following a blank buffer injection of 180 s over the six individual IgG surfaces, five titrated concentrations of antigen in 2-fold dilutions were simultaneously injected in the horizontal direction. The binding interactions were monitored over a 10-min association period and a 45-min dissociation period using a high flow rate of 40 μl/min. The last channel was injected with PBS/Tween/EDTA for reference subtraction. The surfaces were regenerated with two 18-s pulses of glycine, pH 1.5, at 100 μl/min in both horizontal and vertical directions to allow the capture of different serum IgGs for kinetic binding measurements.

##### Data Analysis

The collected sensorgram data were processed by reference subtraction using inter-spots in addition to double-referencing with the in-line buffer blank. The integrated ProteOn Manager software version 3.1.0.6 was used to fit the data with the Langmuir model describing a 1:1 binding stoichiometry. *k_a_* is the association rate constant for the antibody-antigen binding; *k_d_* is the dissociation rate constant of the antibody-target complex, and *K_D_* is the equilibrium dissociation constant, defined by the *k_d_*/*k_a_* ratio. In addition to determining the apparent binding affinity from the fitted binding curves, the experimental binding capacity of the surface, *R*_max_, was also derived. A comparison of the experimental *R*_max_ and theoretical *R*_max_ provides the percentage of IgGs that are specific to the antigen among the total IgGs present in the serum (Equation 1). To obtain the theoretical *R*_max_, Equation 2 was used.

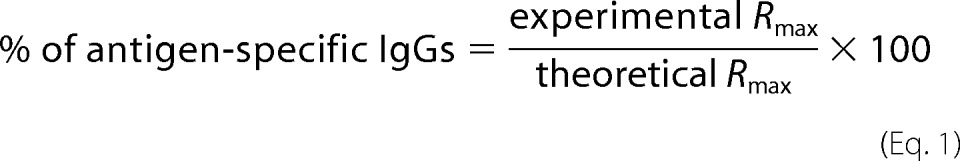


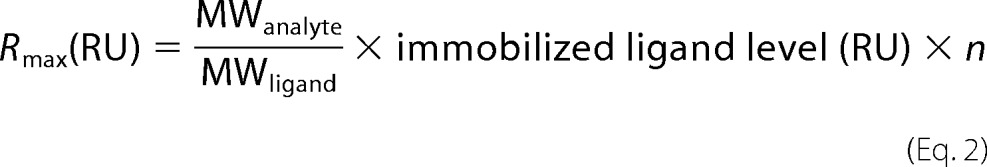
 where MW_analyte_ is the molecular weight of the antigen; MW_ligand_ is the molecular weight of IgGs captured on the sensor surface, and *n* is the binding stoichiometry of the reaction. The immobilized ligand level is the level of captured serum IgGs, which includes both endogenous IgGs and antigen-specific IgGs.

### Epitope Mapping by HDX LC/MS

#### 

##### Sample Preparation

To prepare samples for epitope mapping analysis by mass spectrometry, a diluted sample (∼2 ml) of each purified mouse IgG was concentrated using a 100,000 molecular weight cutoff centrifugal filter device (Millipore, Billerica, MA). The loaded filter devices were spun in a swinging bucket rotor at 4000 × *g* for 40 min at 4 °C. Concentrates of ∼50 μl were collected and subsequently buffer-exchanged with PBS using Zeba spin desalting columns (ThermoFisher Scientific) to maintain a physiological pH for the binding interactions and to ensure consistent deuterium exchange.

To determine the minimal amount of antigen needed for full sequence coverage, different amounts of antigen (4-, 2-, and 1-μl aliquots) were diluted in PBS to a total volume of 35 μl. After analysis, the lowest amount of antigen that yielded the highest sequence coverage was selected and that amount was then directly added to the purified antibody sample to yield a total volume of 35 μl.

##### Deuterium Exchange

Using the H*/*d-X PAL^TM^ robotic system (LEAP Technologies, Inc.), samples containing IgGs incubated with human IL-13 were added to a D_2_O-containing buffer, and the reactions were subsequently quenched at consistent times and temperatures using an automated sample run list. Two separate sample compartments were used for each experiment. One compartment was kept at 20 °C for D_2_O labeling (deuterium exchange), and the other was maintained at 4 °C for reaction quenching. After the samples were prepared, they were transferred into Chromacol screen top vials (ThermoFisher Scientific) and stored in individual positions within the 4 °C sample compartments. A work list was written using HDxDirector (version 1.0.4.0) with the following steps: 1) dilution and mixing of 8 μl of sample with 80 μl of deuterium exchange buffer (10 mm NaH_2_PO_4_ in D_2_O, pH 7.4), and 2) incubation of the mixture at 20 °C for various time periods (60, 120, and 240 s), during which the exchange reaction took place. At the end of each incubation period, 80 μl of each mixture was transferred to a vial at 1 °C containing 80 μl of quench solution (4 m guanidine hydrochloride, 0.5 m tris(2-carboxyethyl)phosphine hydrochloride) and mixed thoroughly. Finally, 60 μl of each quenched reaction mixture was injected onto a Poroszyme® immobilized pepsin column (Life Technologies, Inc.) for 2 min, where the proteins were digested and subsequently desalted on an ACQUITY UPLC BEH C18 Vanguard Pre-column (Waters Technologies) for an additional 2 min prior to injection into a BEH C18 column (Waters Technologies) for LC reverse phase separation.

##### LC/MS System Setup

After the digested peptides were injected onto the C18 column inside the column/valve 4 °C temperature-controlled compartment, a gradient solvent system consisting of precooled mobile phase A (0.1% formic acid, 99% HPLC water, 1% acetonitrile) and mobile phase B (0.1% formic acid, 5% HPLC water, 95% acetonitrile) was used. The C18 column was first equilibrated at 0% B during the 2-min on-column pepsin digest and then the gradient was moved to 10% B during the 2-min peptide wash on the Vanguard pre-column. The chromatographic separation was performed at 4 °C at a flow rate of 180 μl/min by applying the gradient of mobile phase B from 10% at 0 min to 50% at 6 min to 90% at 6.5–7.5 min and finally to 0% at 8–10 min. After chromatographic separation, the sample entered the Orbitrap Fusion^TM^ mass spectrometer operated in positive electrospray ionization mode. The employed method included activated types of collision-induced dissociation and electron transfer dissociation when identifying control peptides, using a resolution of 120,000, a minimum signal of 5000, an isolation width of 3.0, and a normalized collision energy of 30.0 V. The S-lens radiofrequency (rf) level was set at 60%. For peptide identification, the data were collected in profile mode for the full MS scan and in centroid mode for the collision-induced dissociation and electron transfer dissociation MS/MS scans. The data were collected over a mass range of 350–1800 Da. For deuterated samples, no MS/MS data were collected.

##### Data Processing

The collected raw LC-MS/MS fragmentation data from the pepsin digest were analyzed using various software tools for peptide identification. The fragmentation data of the control sample (antigen in the absence of IgGs) were analyzed using Proteome Discover 1.4 (Thermo Scientific) and PMi Byonic (Protein Metrics, Inc.) and compared with the given sequence to generate a list of peptides and retention times. Raw LC-MS/MS data files were preprocessed and converted to ASCII format using proprietary in-house software. The identified peptides were then matched and summarized. The epitopes were determined by calculating the differences in average mass shifting induced by the protection of the region from deuterium labeling.

## Author Contributions

D. Y. wrote the first draft of the manuscript. D. Y. and L. F. developed various methods, performed the experiments, and analyzed the data. M. L. evaluated the peptide microarray and facilitated the implementation of the methods. K. T. provided HDX LC/MS experimental support and software for data analysis. R. K. provided technical guidance and oversight throughout the entire process. S. S. conceptualized the process and provided oversight. All authors discussed the results and implications and commented on the manuscript at all stages.

## Footnotes

2 The abbreviations used are:

SPR: surface plasmon resonance
QAR: quality of antibody response
RU: response unit
MEA: micro-extractor automated instrument
HDX: hydrogen deuterium exchange
AUC: analytical ultracentrifugation
cyno: cynomolgus
BisTris: 2-[bis(2-hydroxyethyl)amino]-2-(hydroxymethyl)propane-1,3-diol
BAFF: B-cell activating factor.

## References

[1] Köhler G. (1985) Derivation and diversification of monoclonal antibodies. EMBO J. 4, 1359–1365 3928369 10.1002/j.1460-2075.1985.tb03787.x PMC554352

[2] Strebhardt K., and Ullrich A. (2008) Paul Ehrlich's magic bullet concept: 100 years of progress. Nat. Rev. Cancer 8, 473–480 18469827 10.1038/nrc2394

[3] An Z. (2010) Monoclonal antibodies–a proven and rapidly expanding therapeutic modality for human diseases. Protein Cell 1, 319–330 21203944 10.1007/s13238-010-0052-8 PMC4875100

[4] Chan A. C., and Carter P. J. (2010) Therapeutic antibodies for autoimmunity and inflammation. Nat. Rev. Immunol. 10, 301–316 20414204 10.1038/nri2761

[5] Weiner L. M., Surana R., and Wang S. (2010) Monoclonal antibodies: versatile platforms for cancer immunotherapy. Nat. Rev. Immunol. 10, 317–327 20414205 10.1038/nri2744 PMC3508064

[6] Reichert J. M. (2012) Marketed therapeutic antibodies compendium. MAbs 4, 413–415 22531442 10.4161/mabs.19931 PMC3355480

[7] Deantonio C., Cotella D., Macor P., Santoro C., and Sblattero D. (2014) Phage display technology for human monoclonal antibodies. Methods Mol. Biol. 1060, 277–295 24037846 10.1007/978-1-62703-586-6_14

[8] Traggiai E., Becker S., Subbarao K., Kolesnikova L., Uematsu Y., Gismondo M. R., Murphy B. R., Rappuoli R., and Lanzavecchia A. (2004) An efficient method to make human monoclonal antibodies from memory B cells: potent neutralization of SARS coronavirus. Nat. Med. 10, 871–875 15247913 10.1038/nm1080 PMC7095806

[9] Lee E.-C., Liang Q., Ali H., Bayliss L., Beasley A., Bloomfield-Gerdes T., Bonoli L., Brown R., Campbell J., Carpenter A., Chalk S., Davis A., England N., Fane-Dremucheva A., Franz B., et al (2014) Complete humanization of the mouse immunoglobulin loci enables efficient therapeutic antibody discovery. Nat. Biotechnol. 32, 356–363 24633243 10.1038/nbt.2825

[10] Nossal G. J. (1992) The molecular and cellular basis of affinity maturation in the antibody response. Cell 68, 1–2 1531039 10.1016/0092-8674(92)90198-l

[11] Rajewsky K. (1996) Clonal selection and learning in the antibody system. Nature 381, 751–758 8657279 10.1038/381751a0

[12] Takemori T., Kaji T., Takahashi Y., Shimoda M., and Rajewsky K. (2014) Generation of memory B cells inside and outside germinal centers. Eur. J. Immunol. 44, 1258–1264 24610726 10.1002/eji.201343716

[13] Pulendran B., and Ahmed R. (2006) Translating innate immunity into immunological memory: implications for vaccine development. Cell 124, 849–863 16497593 10.1016/j.cell.2006.02.019

[14] Dimitrov D. S. (2010) Therapeutic antibodies, vaccines and antibodyomes. MAbs 2, 347–356 20400863 10.4161/mabs.2.3.11779 PMC2881260

[15] Hanly W. C., Artwohl J. E., and Bennett B. T. (1995) Review of polyclonal antibody production procedures in mammals and poultry. ILAR J. 37, 93–118 11528030 10.1093/ilar.37.3.93

[16] Leenaars M., and Hendriksen C. F. (2005) Critical steps in the production of polyclonal and monoclonal antibodies: evaluation and recommendations. ILAR J. 46, 269–279 15953834 10.1093/ilar.46.3.269

[17] Burns R. (2009) Immunisation strategies for antibody production. Methods Mol. Biol. 508, 27–35 19301744 10.1007/978-1-59745-062-1_3

[18] Kaufmann S. H., McElrath M. J., Lewis D. J., and Del Giudice G. (2014) Challenges and responses in human vaccine development. Curr. Opin. Immunol. 28, 18–26 24561742 10.1016/j.coi.2014.01.009

[19] Miura K., Orcutt A. C., Muratova O. V., Miller L. H., Saul A., and Long C. A. (2008) Development and characterization of a standardized ELISA including a reference serum on each plate to detect antibodies induced by experimental malaria vaccines. Vaccine 26, 193–200 18054414 10.1016/j.vaccine.2007.10.064 PMC2253722

[20] Gan S. D., and Patel K. R. (2013) Enzyme immunoassay and enzyme-linked immunosorbent assay. J. Invest. Dermatol. 133, e12 23949770 10.1038/jid.2013.287

[21] Mori H., Sakamoto O., Xu Q. A., Daikoku M., and Koda A. (1989) Solid phase enzyme-linked immunosorbent assay (ELISA) for anti-sheep erythrocyte antibody in mouse serum. Int. J. Immunopharmacol. 11, 597–606 2681006 10.1016/0192-0561(89)90144-6

[22] Wang H., Griffiths M. N., Burton D. R., and Ghazal P. (2000) Rapid antibody responses by low-dose, single-step, dendritic cell-targeted immunization. Proc. Natl. Acad. Sci. U.S.A. 97, 847–852 10639168 10.1073/pnas.97.2.847 PMC15419

[23] Hauge S., Madhun A., Cox R. J., and Haaheim L. R. (2007) Quality and kinetics of the antibody response in mice after three different low-dose influenza virus vaccination strategies. Clin. Vaccine Immunol. 14, 978–983 17596426 10.1128/CVI.00033-07 PMC2044485

[24] Awasthi S., Lubinski J. M., Shaw C. E., Barrett S. M., Cai M., Wang F., Betts M., Kingsley S., Distefano D. J., Balliet J. W., Flynn J. A., Casimiro D. R., Bryan J. T., and Friedman H. M. (2011) Immunization with a vaccine combining herpes simplex virus 2 (HSV-2) glycoprotein C (gC) and gD subunits improves the protection of dorsal root ganglia in mice and reduces the frequency of recurrent vaginal shedding of HSV-2 DNA in guinea pigs compared to immunization with gD alone. J. Virol. 85, 10472–10486 21813597 10.1128/JVI.00849-11 PMC3187515

[25] McDonnell J. M. (2001) Surface plasmon resonance: towards an understanding of the mechanisms of biological molecular recognition. Curr. Opin. Chem. Biol. 5, 572–577 11578932 10.1016/s1367-5931(00)00251-9

[26] Hoa X. D., Kirk A. G., and Tabrizian M. (2007) Towards integrated and sensitive surface plasmon resonance biosensors: a review of recent progress. Biosens. Bioelectron. 23, 151–160 17716889 10.1016/j.bios.2007.07.001

[27] Heinrich L., Tissot N., Hartmann D. J., and Cohen R. (2010) Comparison of the results obtained by ELISA and surface plasmon resonance for the determination of antibody affinity. J. Immunol. Methods 352, 13–22 19854197 10.1016/j.jim.2009.10.002

[28] Campagnolo C., Meyers K. J., Ryan T., Atkinson R. C., Chen Y.-T., Scanlan M. J., Ritter G., Old L. J., and Batt C. A. (2004) Real-time, label-free monitoring of tumor antigen and serum antibody interactions. J. Biochem. Biophys. Methods 61, 283–298 15571777 10.1016/j.jbbm.2004.05.006

[29] Cho H.-S., and Park N.-Y. (2006) Serodiagnostic comparison between two methods, ELISA and surface plasmon resonance for the detection of antibodies of classical swine fever. J. Vet. Med. Sci. 68, 1327–1329 17213702 10.1292/jvms.68.1327

[30] Cho H. S., and Kim T. J. (2007) Comparison of surface plasmon resonance imaging and enzyme-linked immunosorbent assay for the detection of antibodies against iridovirus in rock bream (*Oplegnathus fasciatus*). J. Vet. Diagn. Invest. 19, 414–416 17609354 10.1177/104063870701900414

[31] Obunga V. H., Gelfanova V., Rathmanchalam R., Bailey A., Sloan-Lancaster J., and Huang L. (2009) Determination of the mechanism of action of anti-fast antibody by epitope mapping and homology modeling. Biochemistry (Mosc.) 48, 7251–7260 10.1021/bi900296g 19588926

[32] Kamata T., Handa M., Takakuwa S., Sato Y., Kawai Y., Ikeda Y., and Aiso S. (2013) Epitope mapping for monoclonal antibody reveals the activation mechanism for αVβ3 integrin. PloS One 8, e66096 23840404 10.1371/journal.pone.0066096 PMC3688720

[33] Amit A. G., Mariuzza R. A., Phillips S. E., and Poljak R. J. (1986) Three-dimensional structure of an antigen-antibody complex at 2.8 A resolution. Science 233, 747–753 2426778 10.1126/science.2426778

[34] Benjamin D. C., and Perdue S. S. (1996) Site-directed mutagenesis in epitope mapping. Methods 9, 508–515 8812706 10.1006/meth.1996.0058

[35] Petrakou E., Murray A., Rosamund C., Graves L., and Price M. R. (1998) Evaluation of Pepscan analyses for epitope mapping of anti-MUC1 monoclonal antibodies–a comparative study and review of five antibodies. Anticancer Res. 18, 4419–4421 9891503

[36] Masch A., Zerweck J., Reimer U., Wenschuh H., and Schutkowski M. (2010) Antibody signatures defined by high content peptide microarray analysis. Methods Mol. Biol. 669, 161–172 20857365 10.1007/978-1-60761-845-4_13

[37] Pirrone G. F., Iacob R. E., and Engen J. R. (2015) Applications of hydrogen/deuterium exchange MS from 2012 to 2014. Anal. Chem. 87, 99–118 25398026 10.1021/ac5040242 PMC4287169

[38] LaPorte S. L., Juo Z. S., Vaclavikova J., Colf L. A., Qi X., Heller N. M., Keegan A. D., and Garcia K. C. (2008) Molecular and structural basis of cytokine receptor pleiotropy in the interleukin-4/13 system. Cell 132, 259–272 18243101 10.1016/j.cell.2007.12.030 PMC2265076

[39] Moy F. J., Diblasio E., Wilhelm J., and Powers R. (2001) Solution structure of human IL-13 and implication for receptor binding. J. Mol. Biol. 310, 219–230 11419948 10.1006/jmbi.2001.4764

